# Amalgam restoration or just a deposit? A riveting incidental finding- a case report

**DOI:** 10.1186/s12887-020-02428-8

**Published:** 2020-11-20

**Authors:** Anka Sharma, Vikrant O. Kasat, Amit R. Parate, Anirudh Upmanyu, Jaishri S. Pagare

**Affiliations:** 1 Department of Oral Medicine and Radiology, Government Dental College and Hospital, 431001 Aurangabad, Maharashtra India; 2 Department of Dentistry, Jag Pravesh Chandra Hospital, Shastri Park, New Delhi, India

**Keywords:** Amalgam, Deposit, Graphite, Habits, Case report

## Abstract

**Background:**

Parafunctional oral habits are known to cause deleterious effects on maxillofacial structures. One such effect is traumatic injuries secondary to chewing inanimate objects like pencils. Following trauma, the lead of the pencil has been reported to embed in the soft tissue of the oral cavity, appearing as a grayish pigmentation (graphite tattoo). However, such pigmentation has never been reported in the hard tissue (teeth).

**Case presentation:**

We hereby report an interesting, first of its kind case in a four-year-old female child. She had been misdiagnosed and referred for the management of a carious tooth; which was, in reality, an exogenous deposit.

**Conclusions:**

The authors highlight the impact of comprehensive history taking on arriving at the diagnosis. Counselling of the child and the parents goes a long way in flouting such deleterious habits.

## Background

Habit is an unconsciously performed repetitive action. It is an integral part of maturation in children. These habits can be functional or parafunctional [1]. Functional habits result from repeating a function like nasal breathing, chewing, swallowing, phonation, etc. These habits are healthy and do not interfere with the normal growth and development of the child. On the other hand, parafunctional habits like digit sucking, lip biting, bruxism, and chewing inanimate objects (toys, pencils, chalk) are acquired from non-functional actions [2]. In the long run, persistence of these parafunctional habits has a major impact on the child’s orofacial musculature and jaw growth leading to complications like jaw size discrepancy, malocclusion, and traumatic injuries.

Traumatic injuries of the orofacial region are very common amongst toddlers as they tend to fall easily due to incompletely developed muscular coordination [3]. When a child falls with a foreign object in the mouth, for instance, a pencil, the lead may break and get embedded in the soft tissue causing pigmentation (graphite tattoo). Graphite tattoo of the soft tissue (palate, buccal mucosa) has been reported in the literature, but not very frequently [4]. However, such pigmentation has never been reported in the teeth.

We hereby report an interesting, one of its kind case, where a grayish pigmentation on a molar tooth of a child was misdiagnosed as caries in a dental camp. She was referred to the present institute for restoration where it was again misdiagnosed, not as caries but as an amalgam restoration. The child’s habit of chewing pencils had led to lead (graphite) deposition on one of her teeth, mimicking an amalgam restoration.

## Case presentation

Accompanied by parents, a four-year-old, school-going female child reported to the Department of Oral Medicine and Radiology with a referral card. A dental camp organized in the school had referred her for the management of carious 85. When enquired, she denied any discomfort, food lodgement, pain, or swelling with the tooth. The parents denied any systemic history or previous dental visit/ treatment of the child. They also refuted any deleterious habit.

**Fig. 1:**
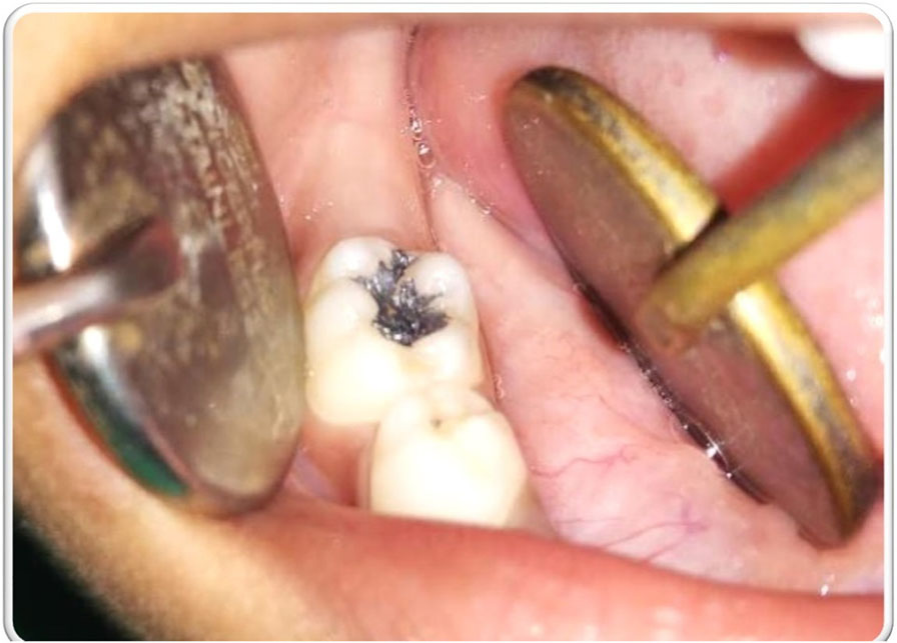
Suspected silver-amalgam restoration on 85

**Fig. 2:**
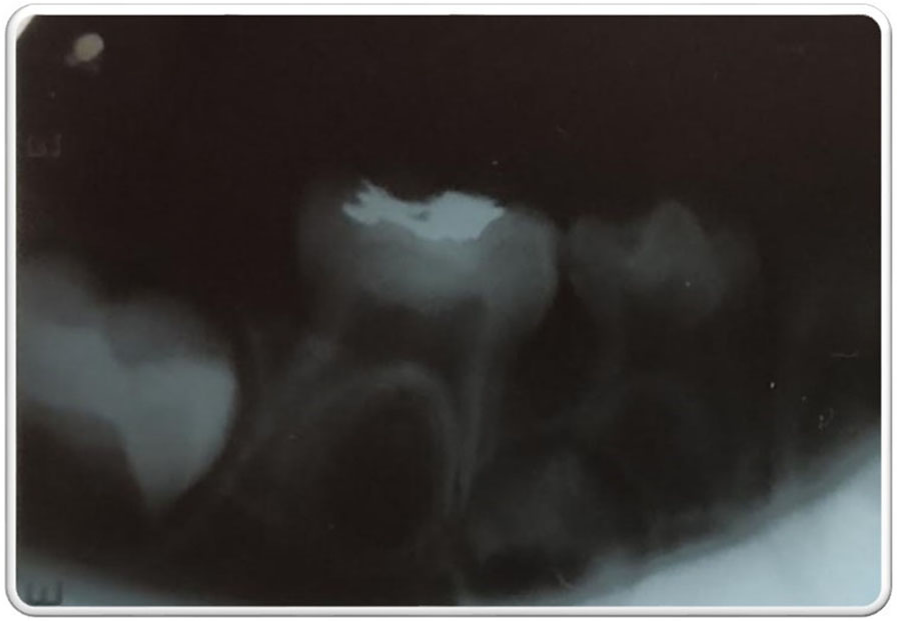
Intra-oral periapical radiograph with mandibular right posterior region showing a well-defined radiopacity on the occlusal aspect of 85. Note that the radiopacity is entirely within the enamel

On inspection, no abnormality was detected extra-orally. Intra-orally, complete set of primary dentition was present. Instead of caries, a class I restoration was noted with 85 (Fig. 1). The tooth was non-tender on percussion and had a healthy periodontium. The rest of the teeth and the soft tissue examination did not reveal any abnormality. An intra-oral periapical radiograph (IOPAR) with 85 revealed a well-defined radiopacity on the occlusal aspect of 85, suggestive of a possible amalgam restoration. The radiopacity, however, was limited entirely to the enamel (Fig. 2), also amalgam restorations are not usually indicated in pediatric patients. This raised suspicion, if the radiopacity was actually an amalgam restoration. Moreover, the patient’s parents had denied any previous dental treatment for the child. This reinforced the suspicion regarding the radiopacity evident with 85.

Hence, the parents were again thoroughly interrogated, upon which the mother recollected that the child had a habit of chewing lead pencils for the past two years and that she whines away from brushing her teeth. The parents assertively claimed this visit to be the patient’s first dental visit.

**Fig. 3:**
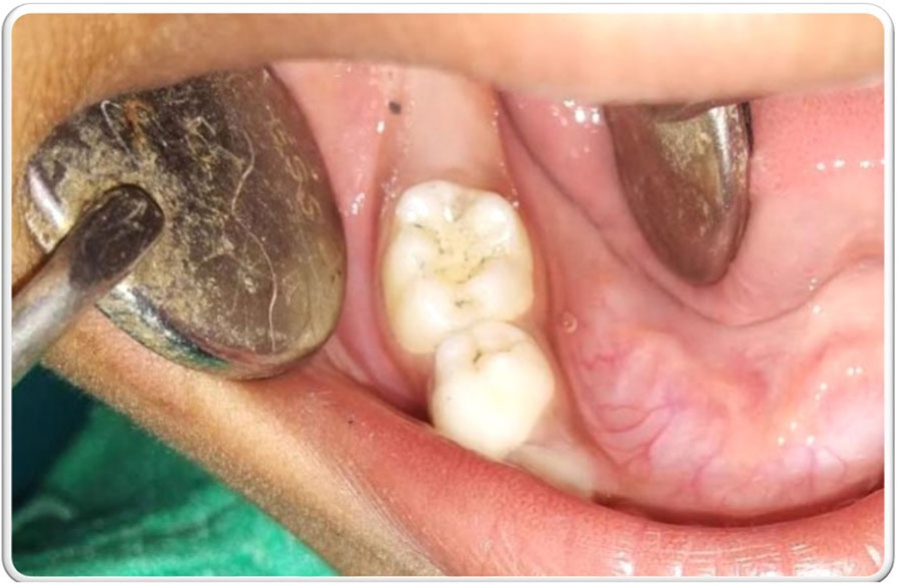
Sound tooth with accentuated pits and fissures evident after removal of the deposit

Keeping in mind these facts, the oral cavity was re-examined carefully. It was noted that all the molars had accentuated pits and fissures. The occlusal surface of 85 was carefully probed, leading to scraping off of some part of the suspected restoration. It was then gently scraped off from the entire occlusal surface, revealing a healthy, non-carious tooth with deep pit and fissures (Fig. 3). The scraping was nothing but, graphite deposit owing to the patient’s habit of chewing lead pencil. Thus, a diagnosis of an exogenous deposit on the tooth mimicking a silver- amalgam restoration was formulated.

The patient was counselled regarding her habit and the parents were explained that how improving the emotional attachment between them and the child could prevent this habit from forming in the first place. Also, the benefits of regular, proper brushing were explained and how inculcating this habit in the child would go a long way in maintaining her overall health.

## Discussion

Oral habits amongst children are influenced by various factors like gender, rank of the child in the family, socioeconomic status, maternal age, and feeding methods [5]. Rondon RG et al., observed increased parafunctional habits in children who were breastfed for less than six months [6]. In order to fulfill their desire for oral stimulation toddlers tend to chew inanimate objects like pencils and toothbrushes [3]. Given the overall lack of muscular coordination at this age, the child often stumbles with these foreign objects in the mouth, sustaining injuries. These injuries range from life-threatening to minor self-healing ones. Aggarwal et al., reported a case where a child fell face-off with a lead pencil in the mouth leading to the development of stroke [7].

The minor traumatic injuries are commonly reported in the anterior hard palate of the children. The graphite of the lead pencil gets embedded in the soft tissue causing a grayish-black discoloration, known as the graphite tattoo [4]. If the injury is not severe, this tattoo might go unnoticed for years, only to be discovered in adulthood during self-examination or during a dental appointment. Graphite tattoo in the older age group requires extensive differential diagnosis to rule out malignancy. In cases where the patient fails to recollect an episode of traumatic injury in childhood, biopsy becomes mandatory for definitive diagnosis [8].

Parafunctional habits are reported to be much more common in children belonging to emotionally unavailable parents or dysfunctional families [1]. These habits not only depict the desire of oral stimulation but also signify the attention-seeking behavior of the children. Goyal A and Bharti K, reported a case where the child developed facets in her teeth owing to pencil chewing habit. The child would get anxious when her mother wasn’t around. In order to cope with the anxiety, she started chewing pencil which led to window shaped facets in her anterior teeth [9]. In the present case, the pencil chewing habit of the child led to graphite deposition in her tooth. The fact that the parents failed to recollect this habit in their child points out their lack of attention towards her. It is imperative to counsel the parents and explain to them the significance of emotional bonding with the child.

In addition to the pencil chewing habit, the child also had a bad habit of avoiding brushing the teeth. Given that her teeth had accentuated grooves and fissures, the graphite kept on accumulating on the occlusal surface of the tooth. This deposit was misdiagnosed as caries by the dentist in the oral camp. He referred the child to the present institute for restoration whereby, it was again misdiagnosed as an amalgam restoration. Only after thorough history taking, clinical examination and radiological investigations, the authors reached the diagnosis of graphite deposit.

## Conclusions

The present case emphasizes the impact of a thorough case history taking (especially the habit aspect) and keen examination in arriving at the diagnosis. The authors want to highlight the possibility of exogenous deposits on the teeth of toddlers and young primary school children attributable to their habits. The parents must be counselled to give their children due emotional support and attention so that they don’t fall prey to such parafunctional habits.

## Data Availability

Not applicable.

## Abbreviation

IOPAR: Intra-oral peri-apical radiograph
